# A new rabbit model of impaired wound healing in an X-ray-irradiated field

**DOI:** 10.1371/journal.pone.0184534

**Published:** 2017-09-08

**Authors:** Kazutoshi Fujita, Soh Nishimoto, Toshihiro Fujiwara, Yohei Sotsuka, Maki Tonooka, Kenichiro Kawai, Masao Kakibuchi

**Affiliations:** Department of Plastic Surgery, Hyogo College of Medicine, Nishinomiya, Hyogo, Japan; National Cheng Kung University, TAIWAN

## Abstract

Radiation is an important therapy for cancer with many benefits; however, its side effects, such as impaired wound healing, are a major problem. While many attempts have been made to overcome this particular disadvantage, there are few effective treatments for impaired wound healing in an X-ray-irradiated field. One reason for this deficiency is the lack of experimental models, especially animal models. We have previously reported a mouse model of impaired wound healing in which the irradiation area was restricted to the hindlimbs. In this mouse model, due to the size of the animal, a diameter of five millimeters was considered the largest wound size suitable for the model. In addition, the transplanted cells had to be harvested from other inbred animals. To investigate larger wounds and the impact of autologous specimen delivery, a rabbit model was developed. Rabbits were kept in a special apparatus to shield the body and hindlimbs while the irradiation field was exposed to radiation. Six weeks after irradiation, a 2 x 2 cm, full-thickness skin defect was made inside the irradiation field. Then, the wound area was observed over time. The wound area after irradiation was larger than that without irradiation at all time points. Both angiogenesis and collagen formation were reduced. For further study, as an example of using this model, the effect of autologous platelet-rich plasma (PRP) was observed. Autologous PRP from peripheral blood (pb-PRP) and bone marrow aspirate (bm-PRP) was processed and injected into the wounds in the irradiated field. Two weeks later, the wounds treated with bm-PRP were significantly smaller than those treated with phosphate buffer vehicle controls. In contrast, the wounds treated with pb-PRP were not significantly different from the controls. This rabbit model is useful for investigating the mechanism of impaired wound healing in an X-ray-irradiated field.

## Introduction

Radiation has become indispensable in clinical practices such as cancer therapy and interventional radiological treatments. In addition, the frequency of compromised healing of irradiated lesions is increasing.[1–3] Despite many clinical efforts, e.g. coverage with vascularized muscular flap[4] or high concentration cytokine administration,[5] the problem has not been solved, yet. The detailed mechanism of impaired wound healing remains poorly understood. One reason for this deficiency is the lack of experimental models, especially animal models. Some animal models have been used to study cutaneous injury caused by irradiation.[6–9] The wound healing that occurs in these models can be attributed to the contraction of surrounding healthy tissue, which is out of the irradiated area. Whereas in clinical situations, wound-healing impairment is observed inside of the irradiated area. There are few animal models for studying wound healing in an irradiated field. Certain levels of whole-body irradiation can be lethal to an animal, and irradiation to vital organs has to be avoided. We have previously reported a mouse model[10] in which the irradiation area was restricted to the hindlimbs. Poor wound healing in the irradiated field and advantages of delivering cultured adipose-derived stromal cells were both observed in this mouse model. However, due to the size of the animal, a diameter of five millimeters was considered the largest suitable wound size. In addition, the transplanted cells had to be harvested from other inbred animals.

To investigate larger wounds and the impact of autologous specimen delivery, a rabbit model was developed. As an example of certifying feasibility of this model, impacts of autologous platelet rich plasma, processed from either peripheral blood or bone marrow aspirate, on the wound in irradiated field was observed macroscopically and histologically.

## Materials and methods

Japanese white rabbits (female, nine weeks old, approximately 2 kg) were obtained from Japan SLC, Inc. (Hamamatsu, Japan). The animals were housed under standard conditions and treated according to the protocol approved by the animal care and use committee of the Hyogo College of Medicine (No. 12–066). The laboratory animal care center complies with the Guide for the Care and Use of Laboratory Animals by the United States National Research Council.[11] The animals were housed individually in cages with dimensions of 35 cm x 53 cm x 35 cm. The temperature and humidity were maintained at 20 ± 2°C and 40–60%, respectively. The ventilation frequency was 15 times per hour. The excreta were automatically washed away. The lighting was 150–300 lx, 40–85 cm above the floor, 12 hours per day. The animals had free access to water and CR-3 forage (CLEA Japan, Tokyo, Japan). The physical condition of the animals was monitored twice a day. According to the protocol, the animals were to be euthanized if they exhibited severe weight loss, decreased appetite or other signs of severe illness. However, no animals were euthanized prior to the endpoints. The number of animals required for this study was calculated based on the principle of the three Rs (i.e., reduction, refinement and replacement) for research animal welfare.

### X-Ray irradiation

**Fig 1:**
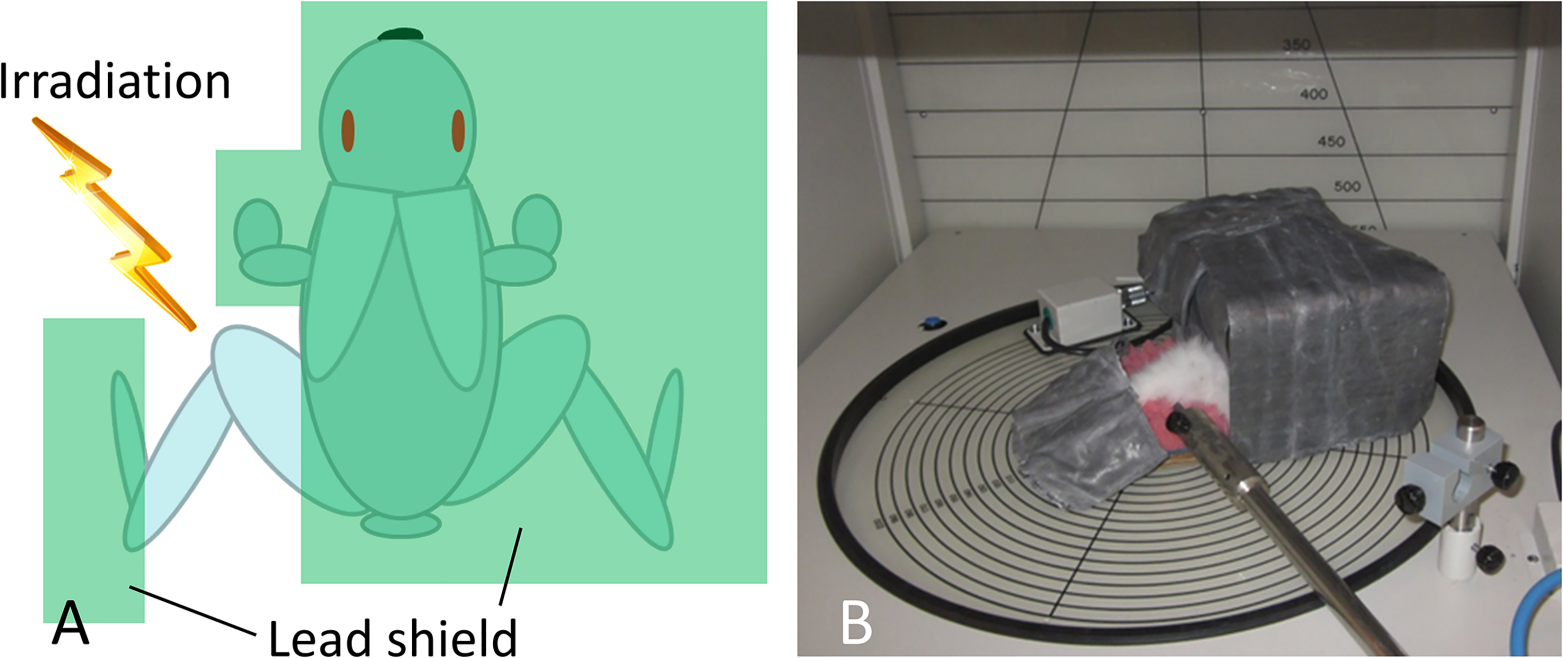
A: Schematic diagram of shielding. Irradiation was restricted to a hindlimb. B: An anesthetized rabbit in a shielding apparatus was placed in an X-ray irradiation unit.

The animals were anesthetized with an intravenous injection of 30 mg/kg pentobarbital sodium through the auricular vein. Then, they were placed in a special apparatus to shield their body with lead. The left hindlimb was placed outside of the apparatus, but the foot distal to the ankle was also shielded. The animal in the apparatus was then placed in an MBR-1520 X-ray irradiation unit (Hitachi Medical, Tokyo Japan), which was operated at 150 kV and 20 mA with a 1.0-mm aluminum filter and a dose rate of 2 Gy/min. The distance from the irradiation source was 350 mm (Fig 1). The duration of time that the animals were placed in the apparatus was within 15 minutes. One rabbit stopped breathing soon after the intravenous administration of anesthetic. All the other animals tolerated this procedure.

### Radiation dose

The radiation dose was determined experimentally, referencing our previous experience in mice.[10] A total of six rabbits, two for each dose, received single doses of 15 Gy, 20 Gy, or 25 Gy irradiation on the left hindlimb. Within three weeks after irradiation, no remarkable changes were observed. After four weeks, hair loss on the irradiated limb was observed in all animals. Spontaneous ulceration was observed in the animals irradiated with 25 Gy. Thus, 20 Gy was determined to be the dose for this study.

### Secondary intervention

Six weeks after irradiation, the rabbits were anesthetized via inhalation analgesia with 3% isoflurane. PRP’s were prepared during the skin defects were created.

### Processing PRP

Peripheral blood and bone marrow aspirate was obtained just prior to creating the skin defects. PRP from peripheral blood and bone marrow aspirate was processed as previously reported.[12] In brief, 2 ml of peripheral blood was drawn from the auricular vein with a 22-gauge elastic catheter and a 5-ml syringe containing 0.5 ml of ACD-A solution (2.2% sodium citrate, 0.8% citrate, 2.2% glucose) (Terumo, Tokyo, Japan) as an anticoagulant. To obtain bone marrow aspirate, the right iliac crest was pierced from the lateral wall in the cranial direction with Komiya’s bone marrow needle (Kurita, Tokyo, Japan), and then bone marrow was aspirated. This technique is much easier than piercing the femur. The aspirate was poured into a 5-ml polypropylene centrifuge tube (Falcon, Corning NY) and spun at 200 g for 10 minutes. The supernatant, including the buffy-coat and the slightly red layer, was aspirated and transferred into other tubes. A secondary centrifugation was performed at 1000 g for 10 minutes. The clear supernatant was aspirated, leaving 250 μl in the tube, which was resuspended to obtain PRP. They were kept on ice until they were injected to the wounds. The numbers of platelets and nucleated cells were counted with an LC-152 veterinary hematology analyzer (Horiba, Kyoto, Japan). These numbers were consistent with those in our previous report.[12]

### Skin defect creation

The hair on both hindlimbs was clipped. A plastic template and a skin-marking pen were used to design a 2 x 2 cm, full-thickness skin defect on the outer side of lower leg, two centimeters distal from the kneecap. The wound was made with a scalpel. An electric cautery was used to stop bleeding.

Three rabbits were used for a series. One each was to receive an injection of pb-PRP (RT+pb-PRP), bm-PRP (RT+bm-PRP) or phosphate-buffered saline (RT+PBS). Injections of 40 μl were performed with a 27-gauge needle into the underlining muscle at each side of the square wound and in the center. The wounds on the right side, which were not irradiated, also received PBS injections (RT-). The animals were monitored during the recovery. For three days after the surgery, 0.2 mg/kg of meloxicam (Boehringer Ingelheim Japan, Tokyo, Japan) was orally administered as an analgesic. Five series of experiments were performed for macroscopic observations. All of the animals tolerated this procedure.

### Observations

The wounds were observed, and they were imaged with a ruler using a digital camera placed parallel to the wounds at a distance of 50 cm. The images were analyzed on a personal computer with ImageJ software (NIH, USA). Skin defect areas were measured by one of the authors, who was blinded to the basis of the images. Epithelial edges were traced with freehand selection, and the framed areas were measured.

### Cell tracking and histological evaluation

Twelve rabbits were used for histological evaluation and for tracking the survivability of injected cells. At the time of wound creation, six weeks after irradiation, skin samples excised from both irradiated and not-irradiated sides were collected for histology.

Cells in the pb-PRP and bm-PRP were rinsed with PBS three times. They were dyed with 5 μg/ml of Cell TrackerTM CM-DiI (Invitrogen, Eugene, OR. USA) fluorescent dye as previously reported.[12] They were suspended in 200 μl of platelet-poor plasma and injected into the wounds on the hindlimbs. Either 2 or 4 weeks after wound creation, the animals were euthanized by an overdose of pentobarbital sodium, and the wounded sites were excised. The specimens were fixed with 10% phosphate-buffered formalin, and paraffin-embedded sections were prepared.

Hematoxylin-eosin staining was performed for gross observations. Histological images were captured using a microscope (PROVIOS AX80, Olympus, Tokyo, Japan) with a 2x objective lens and a digital camera (DP72, Olympus, Tokyo, Japan). Panoramic images were composed with Image Composite Editor (Microsoft, Redmond, WA, USA).

Endothelial cells were immunohistochemically identified with anti-human CD31/PECAM1 antibody (Novus, Littleton, CO. USA), which can cross-react with rabbit endothelial cells. A VECTASTAIN Universal Elite ABC Kit (Vector Laboratories, Burlingame, CA, USA) and diaminobenzidine (DAB substrate, Roche Diagnostics, Dubai, UAE) were used for staining. Hematoxylin was used for counterstaining. Images were captured through a 10x objective lens at random locations in neoplastic granulation tissue (S1 Fig). Image analysis was conducted with ImageJ. Neoplastic granulation area was selected with the freehand selection tool. DAB-stained areas were distinguished with the Colour Deconvolution plugin.[13] A threshold was set to analyze the brown images using bins. The percentage of the positive area was measured with the Analyze Particles function. Fluorescent microscopy was performed to track DiI-labeled cells in sections stained with CD31/PECAM1 and hematoxylin.

Picrosirius red stain (Polysciences, Warrington, PA, USA) was used to observe collagen arrangement. Bright-field and polarized images were captured. The fiber density of neoplastic granulation under the wounds was analyzed using 20x polarized images of random areas. The percentage of colored area in the polarized images was calculated. With the Adobe Photoshop CS2 (Adobe Systems Incorporated, San Jose, CA. USA) Color Range tool, green and yellow areas were selected individually. These images were saved, and the percentages of the selected areas were measured with ImageJ.

### Statistical analysis

The Mann-Whitney U test was applied to determine significant differences in the macroscopic area of non-irradiated and irradiated PBS-treated wounds because the variances of these values were unequal. The paired t-test was applied to compare irradiated PBS-treated and PRP-treated wounds. The bilateral unpaired t-test was used to compare CD31/PECAM1-positive areas among normal and post-irradiated specimens. The Tukey-Kramer test was applied for multiple comparisons in the image analyses.

## Results

### Radiation effects on the skin

During the four weeks after the 20-Gy X-ray irradiation, no particular abnormalities were observed in the animals. After that point, hair loss was observed on the irradiated left hindlimbs. No ulceration was observed at the point of six weeks after the irradiation. Abnormal weight loss and gait deficits were not observed throughout the experiment.

**Fig 2:**
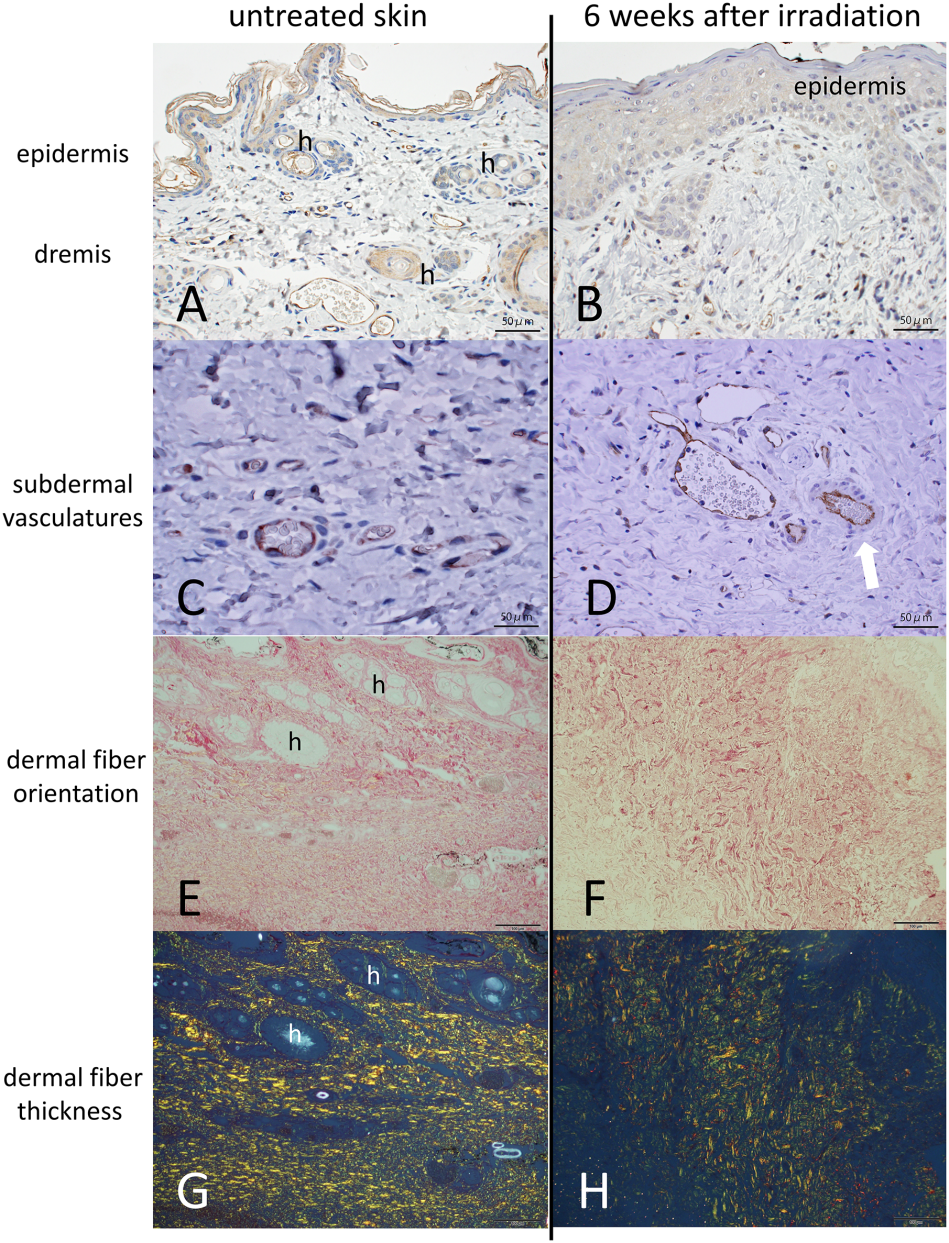
Histological evaluation of untreated rabbit skin and rabbit skin 6 weeks after 25 Gy X-ray irradiation. A, C, E, G (left column): untreated rabbit skin, lower leg. Hair follicles are indicated by “h”. B, D, F, H (right column): 6 weeks after irradiation. A-D: CD31/PECAM1 stained with DAB, counterstained with hematoxylin; 40x objective lens; bar: 50 micro meters. B: Epidermal layer is thick and flat. Appendages are decreased. D: Edema around arterioles (white arrow) and venule dilatation can be observed. E, F: Picrosirius red staining of dermis, bright-field view; 20x objective lens; bar: 100 micro meters. Epidermis is oriented in upper direction. G, H: Polarized view of the same field as E, F. F: Arrangement of collagen fibers is altered. H: Yellow fibers are decreased. See also Table 1.

Histologically (Fig 2), thickening and flattening of the epidermal layer and hair loss were observed in 6-week-post-radiation tissue. Endothelial cells were stained with anti-CD31/PECAM1 antibody; edema around arterioles and venular dilatation were observed. Microvascular density was decreased in irradiated dermis. Picrosirius red staining revealed disrupted arrangements of collagen fibers. In non-treated dermis, fibers school in horizontal orientation. Whereas, fibers jumble in irradiated dermis. The area of thick yellow fibers was lower in the polarized images. The image analysis results (Table 1) indicated that the area of fine green fibers did not change significantly, while the yellow area decreased. As a consequence, total fiber (green + yellow) area and yellow green ratio decreased.

**Table 1:** Percentage of colored areas in picrosirius red-stained, polarized images of normal and post-irradiated skin.

	no treatment	6 weeks post irradiation	p value
green (%)	8.95 ± 1.22	6.60 ± 0.80	0.14
yellow (%)	10.89 ± 0.71	3.32 ± 0.26	<0.01
green+yellow (%)	19.84 ± 1.57	9.92 ± 0.83	<0.01
yellow/green	1.30 ± 0.19	0.53 ± 0.07	<0.01

The values are shown as the mean ± standard error of the mean; p values were calculated with the bilateral t-test. Green areas indicate fine fibers; yellow areas indicate thick fibers. Green + yellow areas indicate total fiber areas. Yellow green ratio indicates how thick those fibers are.

### Wound-healing study

The numbers of injected nucleated cells and platelets for RT+pb-PRP were 64 ± 13 x10^6^ (mean ± standard error of the mean) and 22 ± 4.2 x10^6^, respectively, and those for RT+bm-PRP were 76 ± 6.0 x10^6^ and 48 ± 10 x10^6^, respectively.

**Fig 3:**
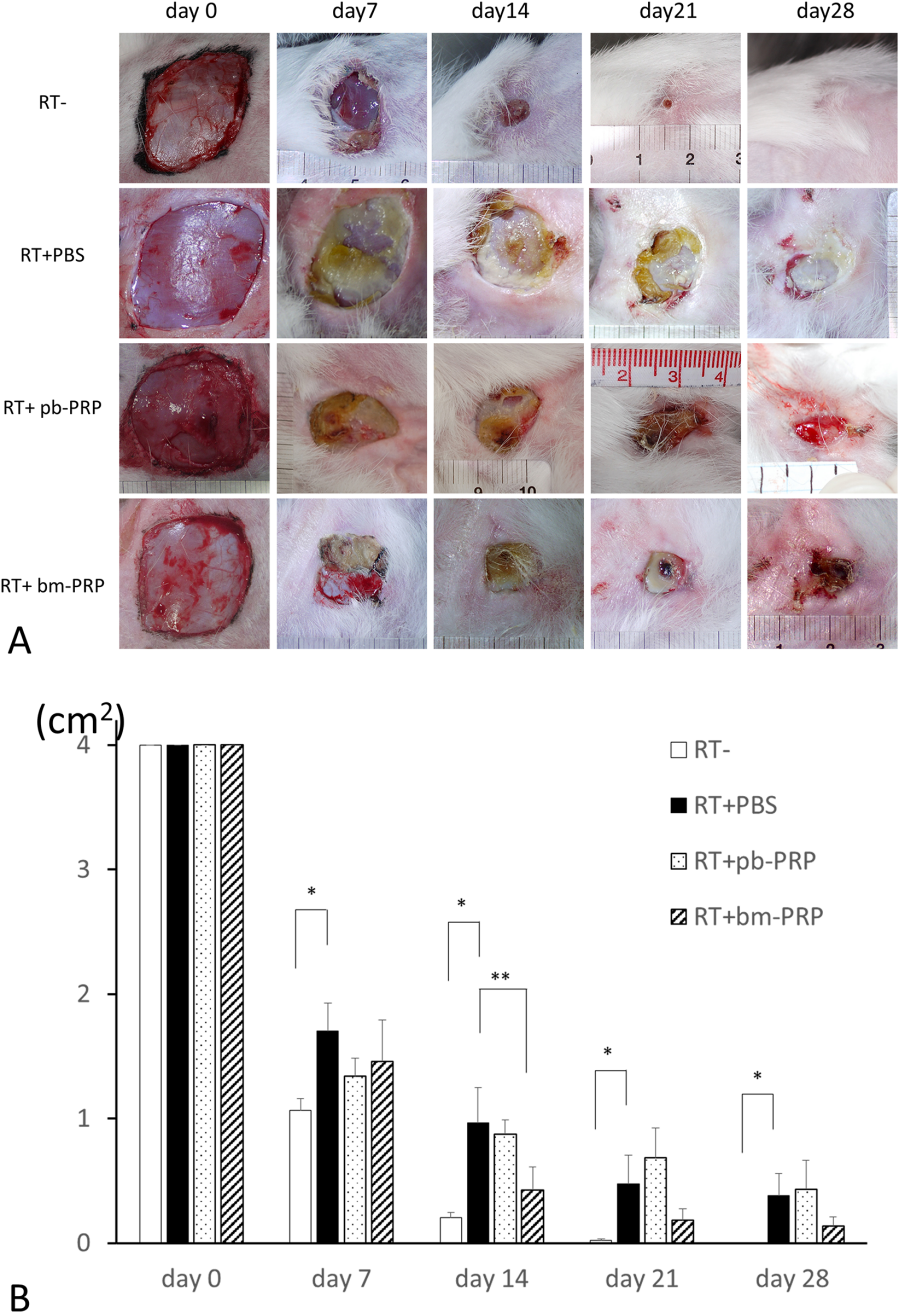
RT-: Wound on non-irradiated limb. RT+PBS: Phosphate buffered saline treated wound on irradiated limb. RT+pb-PRP: Wound on irradiated limb treated with platelet rich plasma derived from peripheral blood. RT+bm-PRP: Wound on irradiated limb treated with bone marrow aspirate derived platelet rich plasma. A: Macroscopic images taken chronographically. Images in horizontal rows are of the same animal. B: Skin defect area in the irradiated field. Error bars: standard error of the mean; *p<0.05, Mann-Whitney test; **p<0.05, paired t-test. The wounds in non-irradiated (RT-) areas were significantly smaller than those in irradiated fields at all time points. RT+bm-PRP-treated wounds were significantly smaller than RT+PBS-treated wounds on day 14.

Chronographically captured images of wounds are shown in Fig 3A. Wound on non-irradiated limb contracted and epithelized almost on day 21. Wounds on irradiated limbs appeared larger than that on non-irradiated limb, at all time points. Yellowish non-vital tissue was observed in wounds on irradiated area. Wound contraction was dominant over epidermal expansion in decreasing wound area in all series.

Measured skin defect areas are shown in Fig 3B, S1 Table. At all time points, the wounds in non-irradiated (RT-) areas were significantly smaller than the wounds in irradiated fields. At 14 days after wound creation, RT+bm-PRP-treated wounds were significantly smaller than RT+PBS-treated wounds. However, the area of RT+pb-PRP-treated wounds was not significantly different from that of wounds treated with RT+PBS.

**Fig 4:**
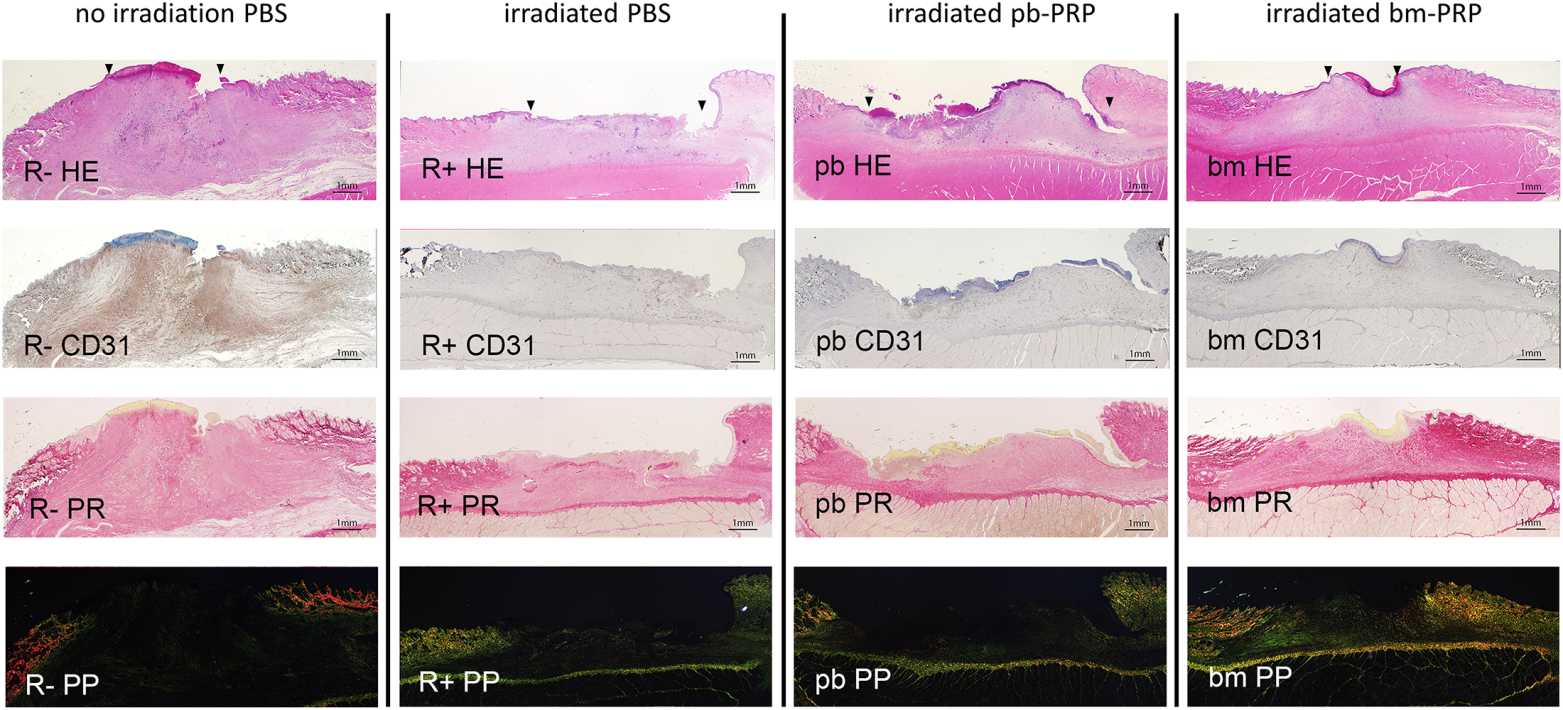
Histological view of the wounds 2 weeks after the surgery. Serial sections were prepared and stained; 2x objective lens; bar: 1 mm. R-: without irradiation, PBS injected. R+: irradiated, PBS injected. pb: irradiated, peripheral blood-derived PRP injected. bm: irradiated, bone marrow aspirate-derived PRP injected. HE: hematoxylin-eosin stain. CD31: DAB stained with anti-CD31/PECAM1 antibody, counterstained with hematoxylin. PR: Picrosirius red stain, bright-field view. PP: Picrosirius red stain, polarized view. Black triangles in first row (HE) indicate epidermal edges.

**Fig 5:**
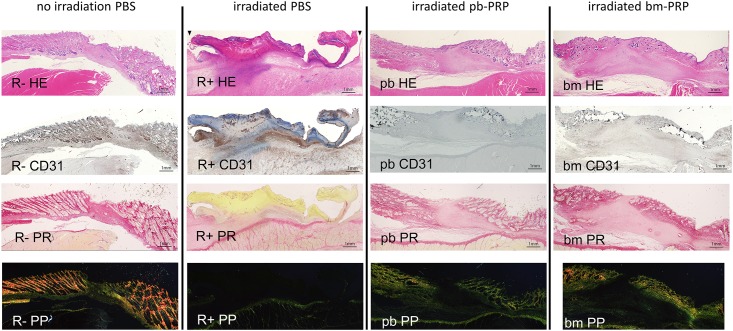
Histological view of the wounds 4 weeks after the surgery. 2x objective lens; bar: 1 mm. R-: without irradiation, PBS injected. R+: irradiated, PBS injected. pb: irradiated, peripheral blood-derived PRP injected. bm: irradiated, bone marrow aspirate-derived PRP injected. HE: hematoxylin-eosin stain. CD31: DAB stained with anti-CD31/PECAM1 antibody, counterstained with hematoxylin. PR: Picrosirius red stain, bright-field view. PP: Picrosirius red stain, polarized view.

The lengths of epidermal tissue defects determined with low-magnification histological sections (Fig 4: 2 weeks post wounding, Fig 5: 4weeks post wounding) correlated with the macroscopic observations. The distance between skin edges with appendages became closer over time. Subcutaneous tissue was thinner in irradiated specimens. In non-radiated wound at 2 weeks, granulation tissue appeared volcano-like cross-section. RT+bm-PRP-treated wound presented this tendency slightly. Irradiated PBS-treated and pb-PRP-treated wounds had flatter granulation. After 4 weeks, RT+PBS-treated wound was covered with non-vital crust. In all irradiated specimens, fiber domain was sparse in granulation tissue.

**Fig 6:**
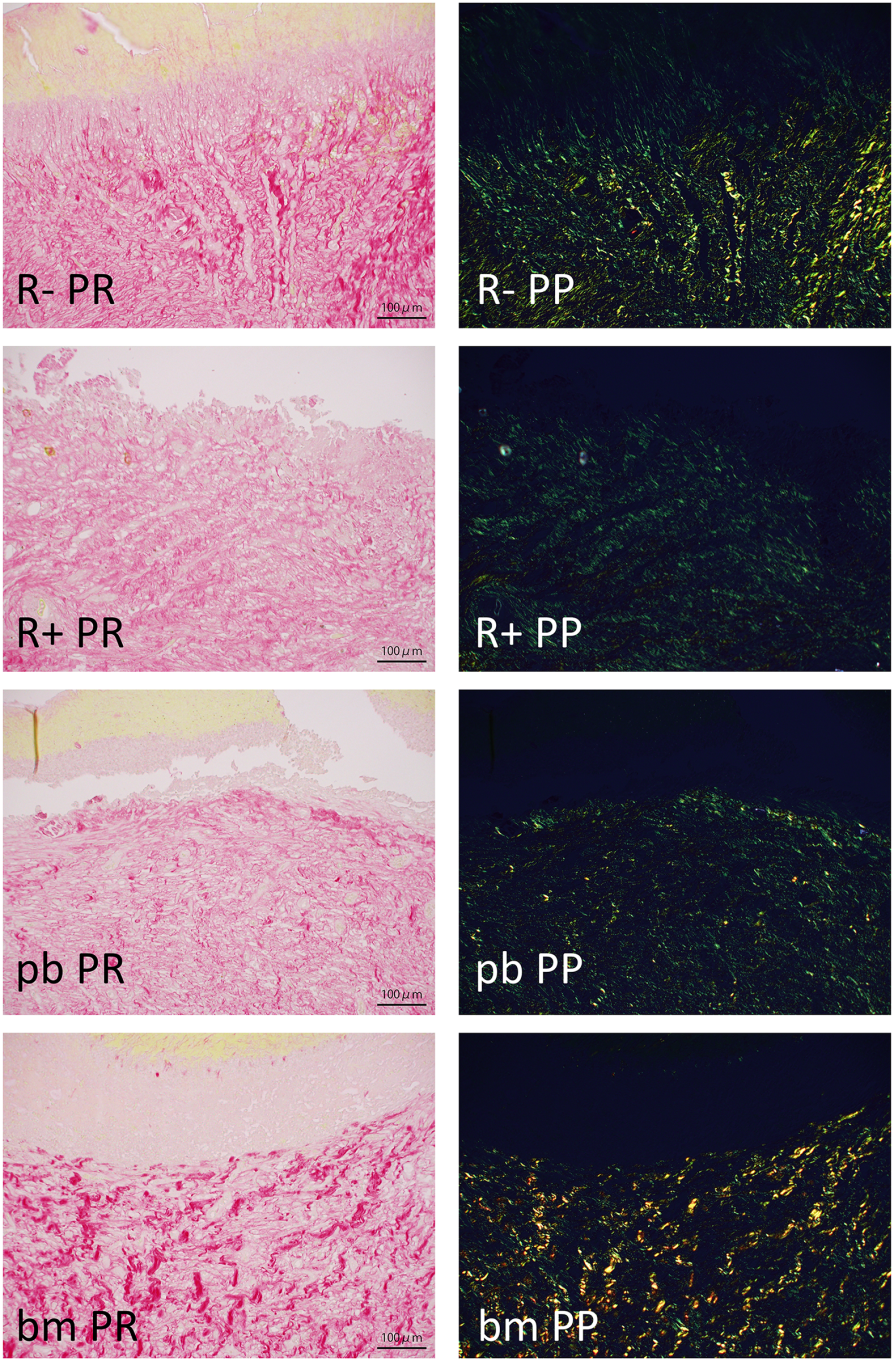
Picrosirius red staining just below epidermal defect. Two weeks after wound creation; 20x objective lens; bar: 100 micro meters. R-: without irradiation, PBS injected. R+: irradiated, PBS injected. pb: irradiated, peripheral blood-derived PRP injected. bm: irradiated, bone marrow aspirate-derived PRP injected. PR: Picrosirius red stain, bright-field view. PP: Picrosirius red stain, polarized view. Green areas indicate fine fibers; yellow areas indicate thick fibers. Marked collagen fiber decrease can be observed in irradiated wounds. See also Table 2.

**Fig 7:**
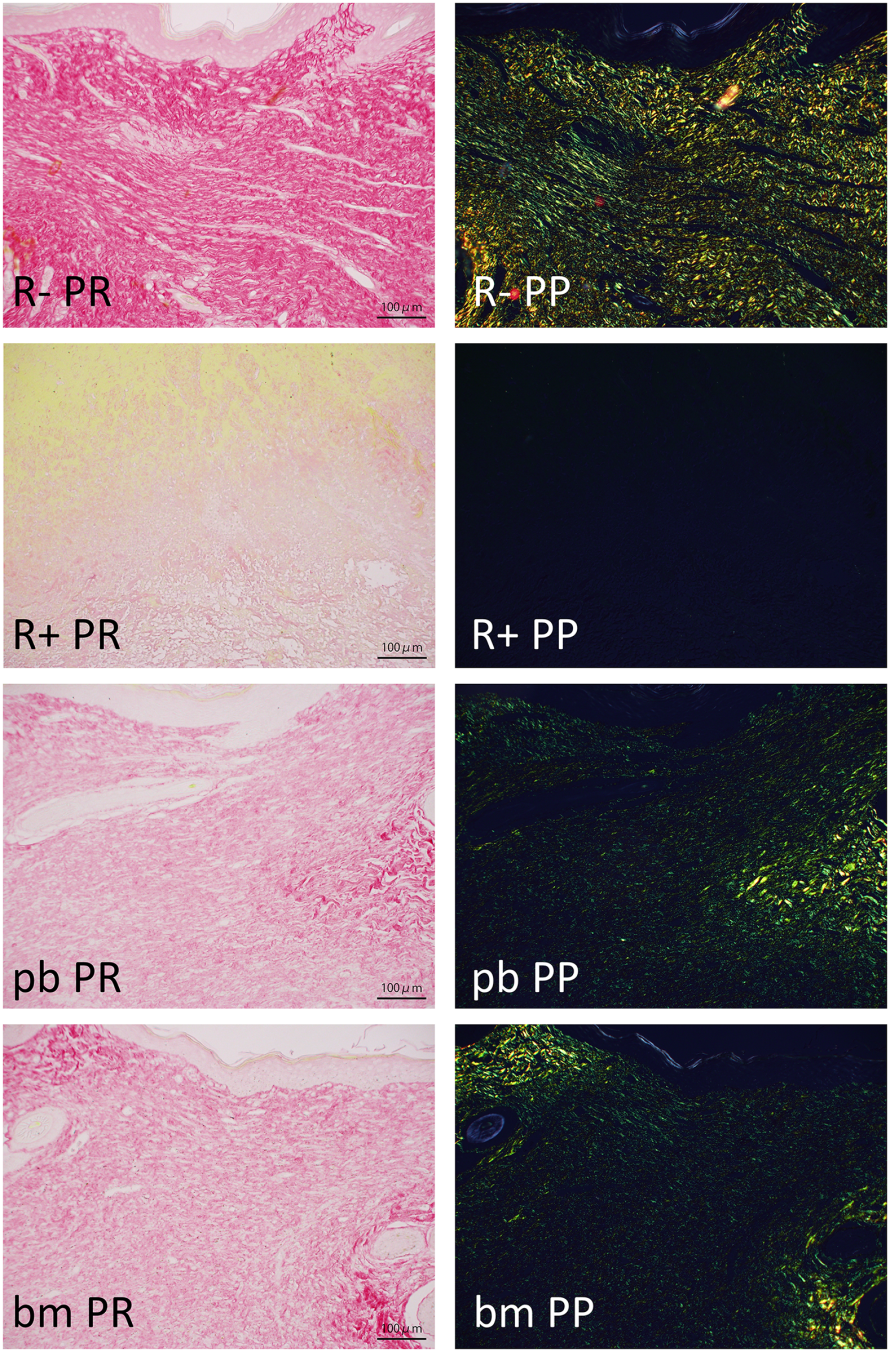
Picrosirius red staining just below epidermal defect. Four weeks after wound creation; 20x objective lens; bar: 100 micro meters. R-: without irradiation, PBS injected. R+: irradiated, PBS injected. pb: irradiated, peripheral blood-derived PRP injected. bm: irradiated, bone marrow aspirate-derived PRP injected. PR: Picrosirius red stain, bright-field view. PP: Picrosirius red stain, polarized view. See also Table 2.

In higher magnification histological sections with picrosirius red staining (Fig 6: 2 weeks post wounding, Fig 7: 4weeks post wounding), especially in RT+PBS-treated specimens, the amount of collagen fibers was apparently decreased; the same result was observed by analyzing polarized images (Table 2). Collagen fiber density was higher in wounds after 2 weeks than after 4 weeks. In two-week specimens, the RT+bm-PRP-treated wounds contained a significantly higher density of yellow fibers compared with that of the RT+PBS-treated controls. This difference was not observed in 4-week specimens.

**Table 2:** Percentage of colored areas in picrosirius red-stained polarized images.

		RT-	RT+PBS	RT+pb-PRP	RT+bm-PRP
2 weeks	green	25.28 ± 1.02 a	2.03 ± 0.22	5.26 ± 0.24 b	3.44 ± 0.34
	yellow	15.59 ± 2.55 a	0.08 ± 0.02	0.42 ± 0.04	5.41 ± 0.28 b
	green+yellow	40.87 ± 2.32 a	2.10 ± 0.23	5.68 ± 0.20	8.85 ± 0.48 b
	yellow/green	0.62 ± 0.12 a	0.04 ± 0.01	0.08 ± 0.01	1.63 ± 0.18 a
4 weeks	green	10.46 ± 0.77 a	0.00 ± 0.00	1.49 ± 0.21	0.59 ± 0.06
	yellow	10.69 ± 2.32 a	0.00 ± 0.00	0.02 ± 0.00	0.01 ± 0.00
	green+yellow	21.15 ± 2.90 a	0.00 ± 0.00	1.52 ± 0.21	0.59 ± 0.05
	yellow/green	1.01 ± 0.16 a	0.16 ± 0.16	0.02 ± 0.00	0.01 ± 0.01

Values are presented as the mean ± standard error of the mean;

^a^: p<0.01 to all other groups,

^b^: p<0.01 to RT+PBS; Tukey-Kramer test.

RT-: without radiation, PBS injected. RT+PBS: irradiated, PBS injected. RT+pb-PRP: irradiated, pb-PRP injected. RT+bm-PRP: irradiated, bm-PRP injected.

The image analysis of CD31/PECAM1-positive area (Table 3) showed a significantly higher vascular density in non-irradiated wounds than in irradiated wounds. In two-week specimens, vascular density was significantly higher in RT+pb-PRP- and RT+bm-PRP-treated wounds than in the PBS-injected counterparts. No significant differences were observed in vascular density among 4-week irradiated specimens.

**Table 3:** Percentage of CD31/PECAM1-positive area in neoplastic granulation tissue.

	RT-	RT+PBS	RT+pb-PRP	RT+bm-PRP
2 weeks	30.99 ± 2.05 a	1.34 ± 0.09	2.97 ± 0.16 b	2.87± 0.28 b
4 weeks	22.79 ± 2.53 a	1.009 ± 0.08	1.39 ± 0.15	0.96± 0.11

(a: p<0.01 to all other groups, b: p<0.01 to RT+PBS; Tukey-Kramer test) RT-: without radiation, PBS injected. RT+PBS: irradiated, PBS injected. RT+pb-PRP: irradiated, pb-PRP injected. RT+bm-PRP: irradiated, bm-PRP injected.

### Cell tracking

**Fig 8:**
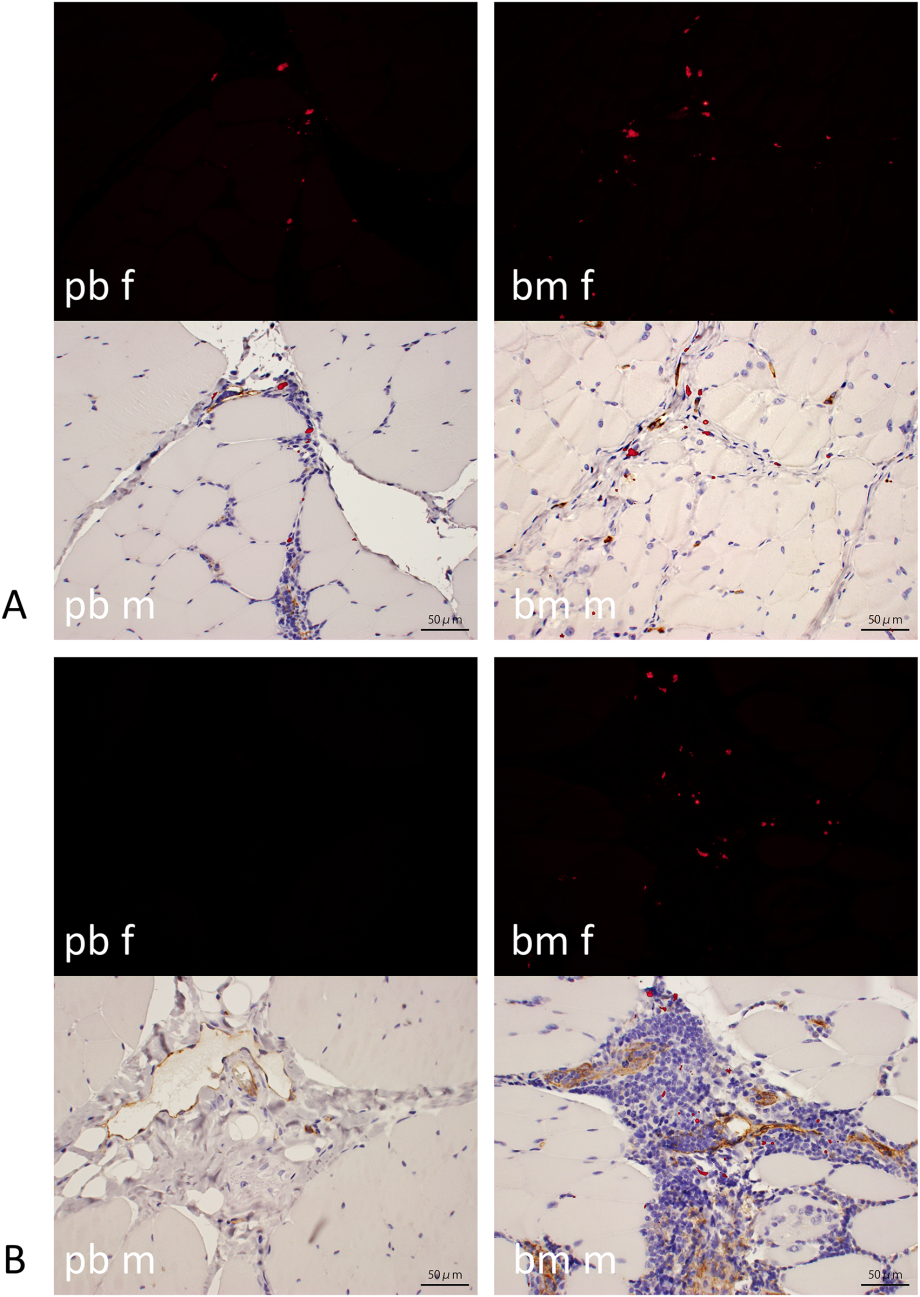
Tracking of implanted cells. DiI-labeled cells were fluorescently identified (red) by CD31/PECAM1 in sections stained with DAB and counterstained with hematoxylin. Objective lens: 40x; bar: 50 micro meters. pb: pb-PRP injected, bm: bm-PRP injected, f: fluorescent view, m: merged with bright-field view. A: 2 weeks after injection. In both pb and bm, fluorescence was identified in wound bed muscular layer. B: 4 weeks after injection. In pb, fluorescence was hardly observed, whereas in bm, robust fluorescence was confirmed. Correlation between fluorescence and DAB stain was unclear.

The cells implanted by PRP injection into the irradiated wound beds were identified in paraffin-embedded sections that were immunohistochemically stained for CD31/PECAM1 (Fig 8). At two weeks after wounds received pb-PRP or bm-PRP, DiI-labeled cells were observed in some cell nests that had formed in the intermuscular bundle space. Most of the fluorescently positive cells were not positive for CD31/PECAM1. After four weeks, fluorescent signals were barely present in RT+pb-PRP-treated wounds. In RT+bm-PRP-treated wounds, robust signals were observed over four weeks. No signals were observed in areas of neoplastic granulation.

## Discussion

The application of radiological imaging and intervention has been increasing, and the same lesions often receive repetitive interventions. The effects of radiation accumulate in the irradiated area and last for a long time. Furthermore, the use of oncological radiation therapy has recently been dramatically increasing. Along with the increasing number of radiological cases, the frequency of compromised wound healing in the irradiated field is also increasing. Yet, the detailed mechanism of wound-healing impairment remains unclear. One main reason for this deficit is the lack of appropriate experimental models, especially in vivo models.

Some animal experimental models for investigating irradiation damage have been reported. As certain amounts of whole-body electromagnetic irradiation are lethal, various means of avoiding irradiating vital organs have been attempted. Rifkin et al.[6] designed an athymic rat model that restricted X-ray irradiation to flank skin by pulling and clamping the skin away from the body. Forcheron et al.[7] irradiated lumbar skin with a gated gamma beam perpendicular to the body axis of mini pigs. Kim et al.[8] irradiated pig skin with accelerated electrons, leveraging the characteristic that electron beams or beta-rays are mostly absorbed by shallow tissue layers and do not penetrate tissue layers. Clinically, usage of electron beams or beta-rays are limited to treating superficial lesions. Rodgers et al.[9] used low penetrating X-rays on guinea pigs, that is with the same concept of not letting deep viscera irradiated. In all of those models cited above, skin defects resulted from the irradiation itself, and wound healing was achieved by the surrounding tissue, out of the irradiated field. This situation is rarely observed in clinical practice. Wound-healing impairment is mostly observed within an irradiated field; as such, the surrounding tissue should be also irradiated in models.

Wang et al.[14] irradiated rats with an electron beam and made linear scars on the flank. To investigate wound healing, especially when observing chronic phenomena, the required skin defect area should be considered. Through our experience in mouse model,[10] a diameter of 5 mm was considered the maximum. There has been a rat model using 1 x 1 cm wounds on the dorsum, inside a field of “photon beam” irradiation,[15] however, the “photon beam” described in this study may be an electron beam. As electron beams do not penetrate deeply, the tissue beneath the wound may have been intact. In our X-ray irradiation rabbit model, skin defect areas were made to be 2 x 2 cm wide.

Rabbits are small mammals in the family Leporidae of the order Lagomorpha, in the Glires clade.[16] They are taxonomically close to Rodentia. These animals have a non-aggressive character, so they are easily bred and handled.[17] Autologous bone marrow aspirate can be obtained with ease up to 5 ml.[12] Bone marrow aspirate cannot be obtained from smaller animals without sacrificing them. The rabbit is the largest animal in the group, and they are categorized as a small animal, thus requiring local animal ethical committee approval. On the other hand, larger animals require more elaborate and secured animal research facilities. At the time being, not many antibodies for rabbits can be obtained. An anti-human CD31/PECAM1 antibody was used in this study; it effectively cross-reacted with rabbit endothelial cells. More antibodies that react to rabbit proteins are expected to be available in the future.

One theories that explains late radiation damage is hypoxia and ischemia of the involved tissue. The proliferation of subendothelial connective tissue in small arteries causes marked narrowing and thrombosis of the microvasculature, which is called progressive obliterative endarteritis.[18] In our rabbit model, randomly arranged fine fibers were observed in the dermis at 6 weeks after the irradiation; edema around arterioles and reduced microvascular density were also observed.

Wound-healing impairment in an irradiated field was reproduced in this model. Macroscopically, decreased wound area seemed to be mostly attributable to contraction, and the contribution of epidermal growth seemed minor. Skin defect area was significantly larger in irradiated groups than in the non-irradiated counterparts at all time points. Collagen density in neoplastic granulation areas was significantly decreased in irradiated wounds. Angiogenesis was also decreased.

Oncological radiation therapy takes advantage of the difference in sensitivity between cancer cells, which have a high mitotic capacity, and normal cells, which are differentiated and in a quiescent state. As a side effect, irradiation may also damage cells that are required to play significant roles in wound healing. Direct cellular damage with chromosomal alteration by radiation may further prevent normal replication, which can delay the restoration of damaged tissue.

PRP derived from peripheral blood (i.e., pb-PRP, in this report) is attracting attention as a good source of autologous growth factors.[19] Our group has reported on the use of bone marrow aspirate-derived PRP (bm-PRP)[20] and has reported a comparison of pb-PRP and bm-PRP effects on chronic ischemia wounds.[12] These two types of PRP have equivalent levels of platelets and growth factors. The nucleated cells contained in pb-PRP are mostly lymphocytes and monocytes. In bm-PRP, they are so-called marrow stromal cells, an admixture of various sorts of multi-potential cells. Clinically, we found promising results from bm-PRP delivery during the surgical reconstruction of radiation ulcers.[21]

In a chronic ischemia study,[12] cells in pb-PRP could not be traced for more than two weeks; in contrast, the cells in bm-PRP could be traced for four weeks. These findings correlate with those of this report. For wounds with chronic ischemia, while pb-PRP could not ameliorate impaired wound healing, bm-PRP accelerated wound healing. The same general phenomenon was observed in this study of radiation-impaired wound healing. Histologically, compared to PBS, pb-PRP led to significantly different fine collagen formation and angiogenesis after two weeks. However, there were no significant differences in macroscopic skin defect area. In this model, PRP was delivered at the time of wound creation. The impact of pb-PRP may have been masked by inflammation from the surgical intervention. Zheng et al.[22] reported a positive effect of cultured bone marrow cells on electron beam-induced acute skin damage in rat hindlimbs. In addition, the local administration of culture-expanded bone marrow cells to a radiation burn has been reported to be successful clinically.[23,24] In the present study, the transplanted cells stayed in the muscle layer, where they were injected. There were no signs of these cells migrating or differentiating into the types of cells that directly constitute neoplastic granulation. Paracrine effects[25] seemed to be the main contribution of the transplanted cells. However, in this rabbit hindlimb study, the positive effect of bm-PRP delivery was not as strong as its effect on chronic ischemia. We speculate that there may be a difference in the initial survivability of the injected cells. This approach to enhance wound healing in irradiated field still needs further investigations, including molecular mechanism of radiation-induced impaired wound healing.

### Conclusion

A rabbit model for investigating wound-healing impairment in an X-ray-irradiated field was developed. Both angiogenesis and collagen formation were reduced in the irradiated field. The model was suitable for observing impacts of autologous platelet rich plasma, derived either from peripheral blood and bone marrow aspirate, on wound healing in irradiated fields.

## Supporting information

S1 Fig Representative views of neoplastic granulation, DAB stained with anti-CD31/PECAM1 antibody, counterstained with hematoxylin. Pictures taken with 10x objective lens.
(TIF)

S1 Table 2x2 cm full-thickness skin defect was made on non-irradiated or irradiated limb. Defected area was measured by image analysis. *ave*: average. *stdev*: standard deviation (unit: cm^2^).
(DOCX)
