# Supplementary figures and images for: A new rabbit model of impaired wound healing in an X-ray-irradiated field
Source: PLoS One. 2017 Sep 8;12(9):e0184534. doi: 10.1371/journal.pone.0184534 (PMC5590982; doi:10.1371/journal.pone.0184534)

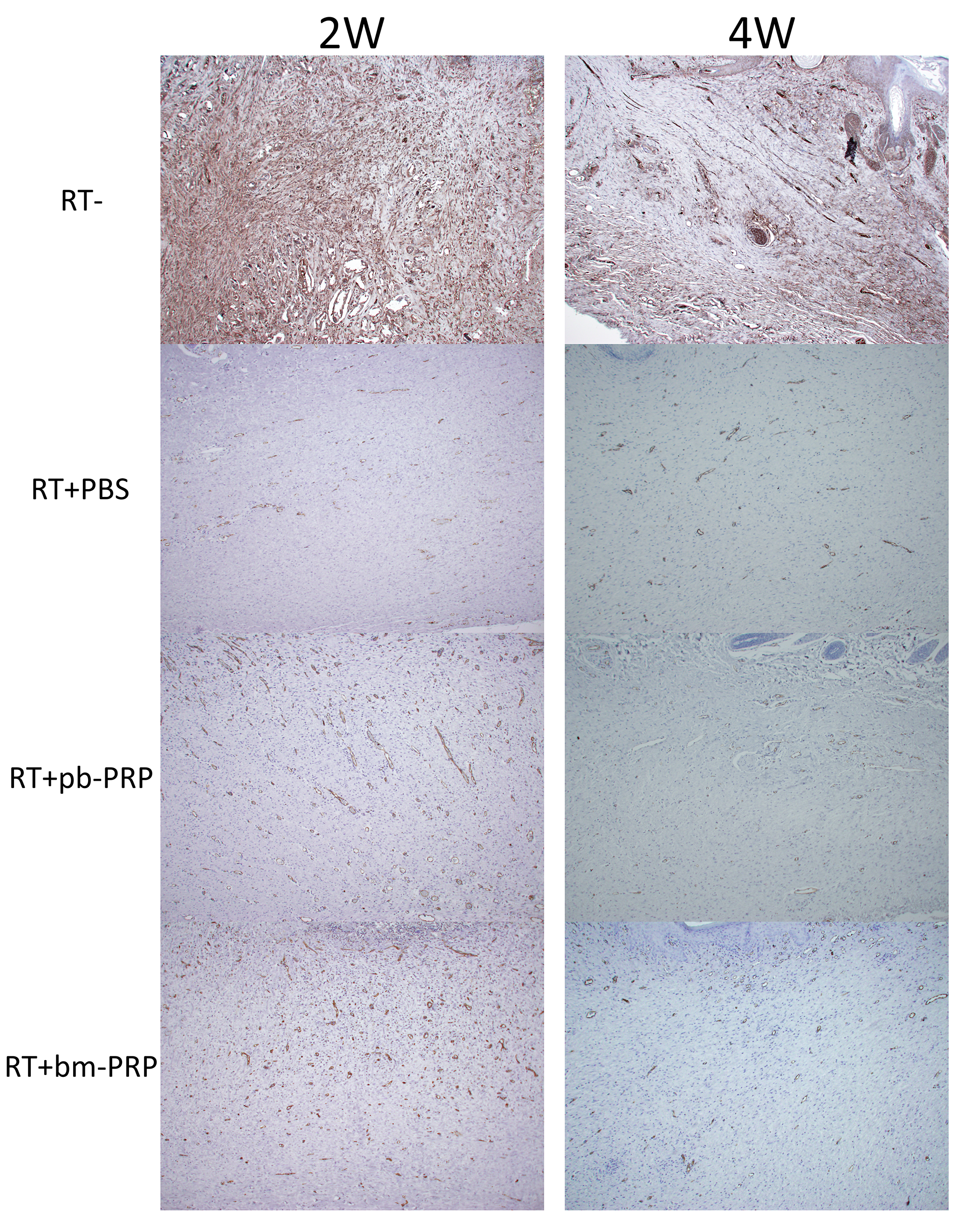

Supplement: S1 Fig — Pictures taken with 10x objective lens. (TIF) [file pone.0184534.s001.tif]
