# Supplementary material for: A new rabbit model of impaired wound healing in an X-ray-irradiated field
Source: PLoS One. 2017 Sep 8;12(9):e0184534. doi: 10.1371/journal.pone.0184534 (PMC5590982; doi:10.1371/journal.pone.0184534)
Supplement: S1 Table — Defected area was measured by image analysis. ave: average. stdev: standard deviation (unit: cm2). (DOCX) [file pone.0184534.s002.docx]

**S1 Table. Skin defect area in the irradiated field after making wounds.**

2x2 cm full-thickness skin defect was made on non-irradiated or irradiated limb. Defected area was measured by image analysis. RT-: no irradiation. RT+PBS: irradiated, phosphate buffered saline injected. RT+pb-PRP: irradiated, peripheral blood derived platelet rich plasma injected. RT+bm-PRP: irradiated, bona marrow aspirate derived platelet rich plasma injected. *ave*: average. *stdev*: standard deviation (unit: cm^2^)

|  |  |  |  |  |  |  |  |  |  |  |  |  |  |  |  |
| --- | --- | --- | --- | --- | --- | --- | --- | --- | --- | --- | --- | --- | --- | --- | --- |
|  | day 0 | | | day 7 | | | day 14 | | | day 21 | | | day 28 | | |
|  | ave | ± | stdev | ave | ± | stdev | ave | ± | stdev | ave | ± | stdev | ave | ± | stdev |
| RT- | 4.00 | ± | 0.00 | 1.06 | ± | 0.35 | 0.20 | ± | 0.15 | 0.02 | ± | 0.05 | 0.00 | ± | 0.01 |
| RT+PBS | 4.00 | ± | 0.00 | 1.70 | ± | 0.45 | 0.96 | ± | 0.57 | 0.48 | ± | 0.46 | 0.38 | ± | 0.35 |
| RT+pb-PRP | 4.00 | ± | 0.00 | 1.34 | ± | 0.29 | 0.87 | ± | 0.23 | 0.68 | ± | 0.48 | 0.43 | ± | 0.46 |
| RT+bm-PRP | 4.00 | ± | 0.00 | 1.46 | ± | 0.66 | 0.43 | ± | 0.37 | 0.18 | ± | 0.18 | 0.14 | ± | 0.14 |
|  |  |  |  |  |  |  |  |  |  |  |  |  |  |  |  |
